# Tailoring Electrocatalytic Properties of sp^2^-Bonded Carbon Nanoforms Through Doping

**DOI:** 10.3390/molecules30061265

**Published:** 2025-03-12

**Authors:** Paweł Szroeder, Agnieszka Banaszak-Piechowska, Ihor Sahalianov

**Affiliations:** 1 Faculty of Physics, Kazimierz Wielki University, Powstańców Wielkopolskich 2, 85-090 Bydgoszcz, Poland; agnb@ukw.edu.pl; 2 Laboratory of Organic Electronics, Department of Science and Technology, Linköping University, SE-60174 Norrköping, Sweden; ihor.sahalianov@liu.se

**Keywords:** graphene, carbon nanotubes, density of π-electron states, heterogeneous electron transfer, intrinsic defects, donor doping, acceptor doping, functionalization

## Abstract

The symmetry of the valence and conduction bands in graphene and carbon nanotubes allows for easy modification of the electronic structure, which is correlated with their electrocatalytic activity. Modifying the electronic structure of the sp^2^-bonded nanocarbons by substituting carbon atoms with electron donors/acceptors and through covalent functionalization can facilitate heterogeneous electron transfer (HET), which is beneficial for designing carbon-based, high-performance electrocatalysts. Based on the Gerischer–Marcus model, we discuss how we can match the density of π-electron states (DOS) of a nanocarbon electrode to the redox potential of redox species using electron and hole doping. Along with the results, this article provides guidance on how to match the properties of nanocarbons to specific electroactive analytes, oxygen reduction reaction (ORR), hydrogen evolution reaction (HER), and oxygen evolution reaction (OER).

## 1. Introduction

An efficient electrocatalyst should be characterized by its ability to lower the energy barrier of desired electrochemical reactions and hinder undesired side reactions. Suitable electrode materials in electrocatalysis should also be low-cost and environmentally friendly. These expectations are met by carbon-based electrocatalysts, which exhibit high activity and strong structure operability [1,2,3,4,5,6,7,8]. Among carbon-based materials, sp^2^-bonded nanocarbons with reduced dimensionality, such as two-dimensional (2D) graphene and one-dimensional (1D) carbon nanotubes (CNTs), have attracted much research attention due to their utility in electronic and structural engineering, which has opened up the possibility of tailoring their properties through lattice deformation, doping, and functionalization [9,10,11,12,13,14].

Low-dimensional sp^2^-bonded carbons combine two features that are key to their use as electrocatalysts: (1) the arrangement of carbon atoms in the form of a two-dimensional, honeycomb-like, crystalline lattice and (2) the electronic properties determined by π-electrons. As for the first feature, carbon atoms in the hexagonal carbon lattice are bound together by strong σ bonds, which provide high chemical stability. In addition, the hexagonal carbon lattice, due to its flexibility, can form planar (graphene), spheroidal (fullerenes, carbon onions), cylindrical (CNTs), and conical (carbon nanocones) structures, whose common feature is a large specific surface area up to 2630 m^2^·g^−1^ [15].

As for the second feature, π-electrons in the hexagonal carbon lattice form a two-dimensional gas of massless Dirac fermions, which flow in the hexagonal plane with a Fermi velocity of 10^6^ m s^−1^ [16]. As a consequence, charge carriers’ mobility reaches a value of $2.5\times 10^{5}\;{cm}^{2}\;V^{-1}\;s^{-1}$, which translates into relatively high in-plane electrical conductivity of $100\;MS\;m^{-1}$ [17]. On the other hand, the in-plane π electrons in unperturbed sp^2^-bonded structures show poor activity in electrocatalytic processes [18]. Therefore, realizing the full application potential of sp^2^-bonded carbon nanoforms in electrocatalysis requires modification of their π-electronic structure, which could stimulate the π-electrons to contribute to the heterogeneous electron transfer (HET). The most obvious strategy is doping, which leads to changes in π-electron or hole concentration. Several approaches have been proposed to dope sp^2^-bonded carbons, which include (i) doping using intrinsic defects [19], (ii) substitutional doping with heteroatoms [20,21], (iii) covalent functionalization [22], and (iv) adsorption-based (noncovalent) doping [23].

Intrinsic defects are the crystalline order perturbations without the presence of foreign atoms. Point defects (vacancies, sp^3^ sites) and line defects (dislocations, edges) have an influence on the electronic structure as they lead to local rehybridization of σ and π bonds [24]. In particular, missing orbitals at the vacancy sites give rise to creating additional midgap bands, which contribute to the density of states (DOS) at the Fermi level [25]. A similar effect is produced by sp^3^ defects, which create π-orbital vacancies [26]. The band structure of the pristine graphene with line defects also possesses flat bands, which lead to the high DOS near the Fermi level [27].

Topological defects, such as n-sided polygons (n≠6), which substitute hexagons in the lattice, induce not only a local curvature of a flat lattice but also produce a finite DOS at the Fermi level that increases with the curvature. In turn, heptagon–pentagon pairs and Stone–Walls defects give rise to the modulation of the DOS [28].

The commonly used substitutional dopants are neighbors of carbon atoms in the periodic table, boron and nitrogen, which produce acceptor-like and donor-like states, respectively [29]. Boron atoms attract delocalized π-electrons from carbon atoms that lead to the lowering of the Fermi level with respect to the Dirac point. The population of the antibonding π* electron states increases, which translates directly into the concentration of holes (p doping) [30]. Nitrogen atoms provide electrons to the conduction band (n doping), which results in the shift of the Fermi level above the Dirac point [31,32].

The π-electronic band structure of graphene functionalized with -OH, -COOH, and -NH_2_ functional groups depends on their configuration on different sites of the sp^2^-bonded carbon lattice. In general, functional groups produce a flat band near the Dirac point and additionally cause a slight down-shift of the Fermi level with respect to the Dirac point (hole doping). However, in some specific configurations, functional groups can open a band gap [33]. Hole doping induced through functionalization is confirmed experimentally in graphene and nanotubes [34,35].

Another method for modifying the electron structure of sp^2^-bonded nanocarbons is molecular-dipole-induced doping. Molecules adsorbed on the surface do not distort the carbon crystalline lattice, but they create high-density electron–hole puddles [36]. Noncovalent doping can be realized through adsorption of strong acceptor (p-doping) or donor (n-doping) molecules [23]. Adsorption of guest molecules on sp^2^-bonded carbons is ubiquitous. On graphene exposed to ambient air, hydrocarbon contaminants adsorb very quickly, which significantly reduces its electrocatalytic activity [37,38]. It has also been shown that molecules of organic dispersants used for the preparation of nanocarbon electrodes adsorb permanently on their surface, giving the doping effect [39].

There are a number of publications in which Fermi level engineering in carbon materials is discussed in terms of electrocatalysis [40,41,42,43,44,45]. In light of all of the existing knowledge, it is an undeniable fact that disorder and doping are necessary to improve the kinetics of the HET on π-electronic carbon systems. In this paper, we present the results of calculating electrode reaction curves on graphene and carbon nanotubes in which the DOS is modified through disorder, heteroatom doping, and functionalization. As redox probes, we use hexacyanoferrate (II)/(III) and hexaammineruthenium (II)/(III), anionic and cationic redox pairs commonly used to assess the electrocatalytic activity of electrode materials. The sp^2^-bonded carbon nanoforms are promising electrode materials in fuel cells and batteries [46], as well as in electrochemical water splitting [47]. Therefore, we also discuss the effect of heteroatom doping and functionalization on the performance of oxygen reduction reaction (ORR), hydrogen evolution reaction (HER), and oxygen evolution reaction (OER). In further parts of the paper, we confront the results obtained with the experimental data available in the literature. In this work, we emphasize Raman spectroscopy, which is an invaluable tool for determining both disorder [48,49,50,51,52] and doping [53,54,55] in carbons.

## 2. Results

### 2.1. Density of π-Electronic States of Electrodes and Alignment of the Redox States

**Figure 1:**
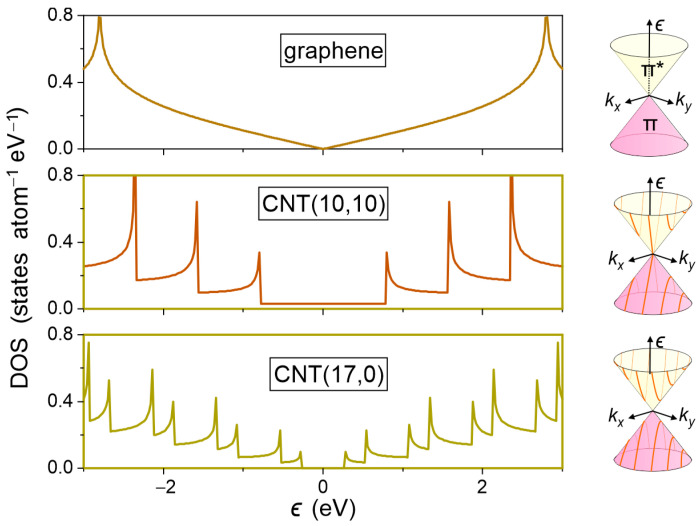
DOS of graphene and a metallic CNT(10,10) and semiconducting carbon nanotube CNT(17,0) calculated using TB approximation. On the right side, Dirac cones for bonding π- and antibonding π*-electronic bands are shown. In CNTs, allowed states in the $\left(k_{x},k_{y}\right)$ space form series of dispersion curves marked as red lines. In metallic CNT, dispersion curves intersect the Dirac point. Zero in the energy scale refers to the Fermi level.

DOS is a key parameter of any electrode material, as it determines the ability to transfer an electron of a certain energy between the electrode and the redox species. Figure 1 shows the DOS of pristine graphene and a metallic and semiconducting carbon nanotube calculated using tight-binding (TB) approximation. On the right side of Figure 1, we show the Dirac cones that occur in the band structure of π-electrons at the vertices of graphene’s first Brillouin zone. A characteristic feature of the DOS of sp^2^-bonded carbons with reduced dimensionality is the occurrence of van Hove singularities, i.e., non-smooth points (kinks and spikes), which originate from critical and saddle points in the electronic band structure.

In 2D graphene, van Hove singularity is formed at ϵ=0, where the apices of Dirac cones formed by bonding π- and antibonding π*-electronic states merge together. At this energy, the kink in the DOS is formed with a density of states equal to zero. The DOS increases smoothly as we move toward higher and lower energies up to a value of ±2.7 eV, where another van Hove singularity in the form of spikes with a high DOS occurs. These sharp maxima correspond to states near the center of the graphene’s first Brillouin zone, whose dispersion relation is flat. Due to the quantum confinement in quasi 1D carbon nanotubes, the allowed states in the $\left(k_{x},k_{y}\right)$ space form a series of one-dimensional sections with corresponding dispersion curves for π and π* electrons, marked in Figure 1. Each curve has a saddle point, which corresponds to the van Hove singularity in the DOS. Thus, a consequence of the limited dimensionality of nanotubes is a larger number of van Hove singularities than in 2D graphene.

While the DOS of a semiconducting CNT(17,0) nanotube at the Fermi level is zero, the DOS of a metallic nanotube is 0.029 states atom^−1^ eV^−1^. For comparison, gold has a DOS at the Fermi level of 0.28 states atom^−1^ eV^−1^, and highly oriented pyrolytic graphite (HOPG) has been reported to have a minimum of 0.0022 states atom^−1^ eV^−1^ [56].

**Figure 2:**
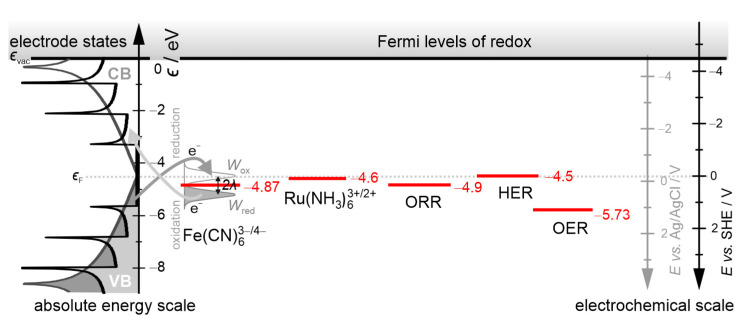
Alignment of the standard reduction potentials of the Fe(CN)_6_^3−^, Ru(NH3)_6_^3+^, ORR, HER, and OER with respect to the position of valence (VB) and conduction (CB) bands of graphene and carbon nanotubes. The E° in V vs. SHE  are converted to absolute energy scale. The $\epsilon_{F,redox}$ values are given in eV vs. vacuum.

We used in the calculations negatively charged Fe(CN)_6_^3−/4−^ and positively charged Ru(NH_3_)_6_^3+/2+^ redox couples, which are commonly used to evaluate the electrocatalytic activity of electrode material. The position of the redox potentials relative to the electron bands of the nanocarbon electrode is shown in Figure 2. The electrochemical scale is converted to the absolute energy scale according to Trassati’s formula: $\epsilon \;\left[eV\right]=-4.5\;eV-eE\;(vs.\;SHE)$ [57]. Unoccupied redox states of the electron acceptor (oxidized form, $W_{ox}(\epsilon )$, and filled states of the electron donor, $W_{red}(\epsilon )$) are separated by doubled reorganization energy, λ. In an anodic reaction, electron transfer occurs from the filled states of the reduced form of the redox couple to empty states of the electrode. In the cathodic reaction, the opposite reaction takes place.

Another calculation concerns the rate of ORR with a four-electron O_2_ reduction pathway: $O_{2}+2H_{2}O+4e^{-}\to 4OH^{-}$. The four-electron pathway ensures high current densities and high onset potentials, which are fundamentally important in air cathodes of fuel cells and metal–air batteries [46]. The standard reduction potential of this reaction is E°=0.402 V vs. SHE [58]. After conversion to the energy scale, we obtain $\epsilon_{F,redox}=-4.902\;eV\;vs.\;vacuum$. In turn, the efficiency of water electrolysis depends on the performance of HER, which occurs on the cathode, and OER occurring on the anode [59]. The cathodic HER pathway is given by $2H^{+}\left(aq\right)+2e^{-}\to H_{2}(g)$ with E°=0.0 V vs. SHE, while the anodic OER occurs according to the pathway $2H_{2}O\;\left(l\right)\to O_{2}\left(g\right)+4H^{+}\left(aq\right)+4e^{-}$ with E°=1.23 V vs. SHE. The standard potentials correspond to $\epsilon_{F,redox}=-4.5\;eV$ and $\epsilon_{F,redox}=-5.73\;eV\;vs.\;vacuum$, respectively. The positions of the ORR, HER, and OER standard potentials relative to the DOS of graphene and CNT are shown in Figure 2.

The Gerischer–Marcus theory states that electron transfer is not limited to states at the Fermi level but occurs between all states forming the quasi-valence and conduction band of the electrode. For this reason, the DOS of π-electrons plays a key role in redox reactions at sp^2^-bonded nanocarbon electrodes.

### 2.2. HET Reaction Rate

#### 2.2.1. Graphene

**Figure 3:**
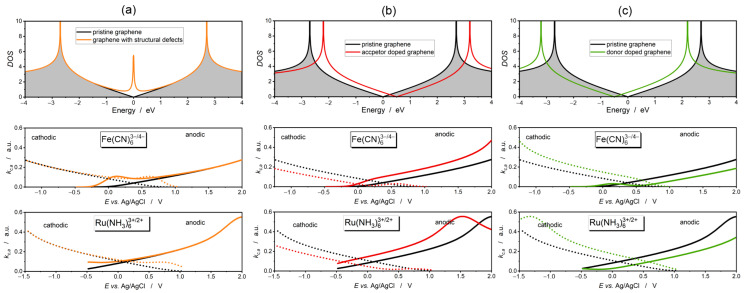
DOS of modified graphene with corresponding cathodic (dotted line) and anodic (continuous line) reaction rate curves for Fe(CN)_6_^3−/4−^ and Ru(NH3)_6_^3+/2+^ redox couples. (**a**) Graphene with structural defects. (**b**) Graphene doped with acceptor dopant; hole concentration of $1.7\times 10^{13}\;{cm}^{-2}$. (**c**) Graphene doped with donor dopant; electron concentration of $1.7\times 10^{13}\;{cm}^{-2}$. The gray color indicates the π-electron states of unmodified graphene. Zero in the energy scale refers to the Fermi level.

In Figure 3, we have juxtaposed the DOS of the modified graphene with simulations of the anodic and cathodic reaction rates calculated based on the Gerischer–Marcus model.

Intrinsic defects, such as graphene edges, vacancies, Stone–Walles defects, etc., induce additional localized states near the Dirac point, which contribute to the DOS of graphene (Figure 3a). The consequence is an increase in the anodic and cathodic reaction rates of the Fe(CN)_6_^3−/4−^ redox couple relative to unmodified graphene at electrode potentials ranging between −0.25 V and 1.0 V vs. Ag/AgCl. The cathodic reaction curve shows a local maximum at the potential of ~+0.5 V vs. Ag/AgCl. A similar maximum is seen in the cathodic curve at the potential of ~+0. 1 V vs. Ag/AgCl. In the case of the Ru(NH3)_6_^3+/2+^ redox couple, a similar effect occurs; however, no clear maxima are seen. This is a consequence of the higher reorganization energy of the Ru(NH_3_)_6_^3+/2+^ redox couple.

Acceptor and donor doping of graphene results in an up-shift and a down-shift of electronic states, respectively. An upward shift of the electron bands on the energy scale caused by acceptor doping causes a downward shift of the anodic and cathodic reaction rate curves (on the electrochemical scale). Donor doping, on the other hand, leads to a shift of the bands toward lower energies. This is accompanied by an upward shift in the cathodic and anodic reaction curves.

#### 2.2.2. Carbon Nanotubes

**Figure 4:**
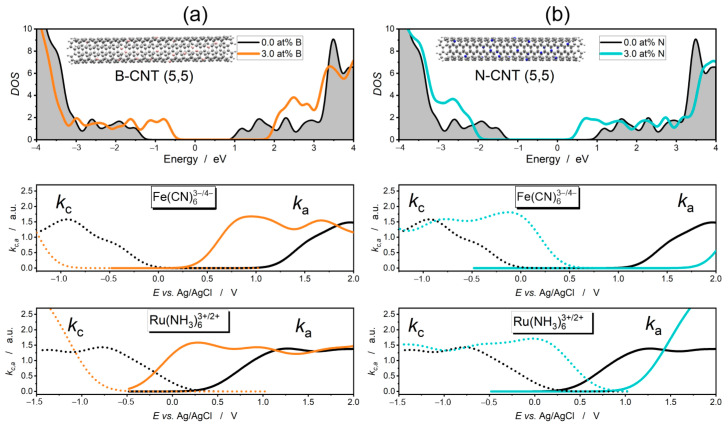
DOS of boron- and nitrogen-doped carbon nanotubes with corresponding cathodic (dotted line) and anodic (continuous line) reaction rate curves for Fe(CN)_6_^3−/4−^ and Ru(NH3)_6_^3+/2+^ redox couples. Atomic concentration of dopants—3 at%. (**a**) B-CNT(5,5). (**b**) N-CNT(5,5). The gray color indicates the π-electron states of unmodified CNT. Zero in the energy scale refers to the Fermi level.

The substitutional boron and nitrogen doping of the nanotubes also significantly affects the shape of the reaction rate curves. As we show in Figure 4, the introduction of boron atoms (electron acceptor) results in the creation of new states within the quasi-band gap below the Fermi level and up-shift of the levels in the quasi-conduction band. At a boron concentration of 3 at%, the bands shift upward by about 1 eV. In contrast, replacing carbon atoms with nitrogen atoms (electron donors) leads to a downward shift of the bands.

As with graphene, the upward shift of the electron bands causes the anodic and cathodic reaction curves to shift significantly to lower potentials, while a downward shift of the electron bands leads to an upward shift of the electrode reaction curves.

**Figure 5:**
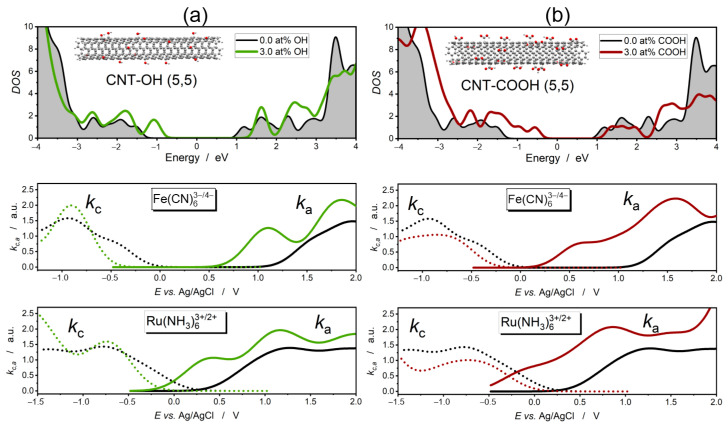
DOS of covalently functionalized carbon nanotubes with corresponding cathodic (dotted line) and anodic (continuous line) reaction rate curves for Fe(CN)_6_^3−/4−^ and Ru(NH3)_6_^3+/2+^ redox couples. Atomic concentration of -OH and -COOH groups—3 at%. (**a**) Hydroxylated CNT(5,5). (**b**) Carboxylated CNT(5,5). The gray color indicates the π-electron states of unmodified CNT. Zero in the energy scale refers to the Fermi level.

Covalent functionalization with -OH and -COOH groups produces an effect similar to the substitution of carbon atoms by acceptor (boron) dopant atoms. An important difference, however, is that functionalization generates bands below the Fermi level, while levels in the quasi-conduction band shift up only slightly. The consequence is a decrease in the quasi-band gap. As shown in Figure 5, carboxyl groups contribute more to the generation of hole states below the Fermi level than hydroxyl groups. This is due to the fact that the -COOH group demonstrates a pronounced electron withdrawal capability compared to -OH due to the presence of two oxygens and stabilization of partial negative charge by carbon atom. The elevated propensity for carboxyl groups to suck electrons out of the sp^2^ carbon lattice is also confirmed by first-principles calculations [60].

Curves of the cathodic reaction rates, $k_{c}$, which reflect the electron transfer from the filled conduction quasi-bands of the functionalized CNTs to empty states of the oxidized form of redox couples, are only slightly shifted toward lower potentials of the electrode. In the case of the carboxylated CNTs, the intensity of $k_{c}$, which is a measure of the cathodic current density during the electrochemical reaction, also decreases. On the other hand, the curves of the anodic reaction rate, $k_{a}$, shift to lower potentials, and their intensities increase significantly due to the abundance of unoccupied states above the top of the valence band produced through functionalization with -OH and -COOH groups. This effect is more spectacular in the case of carboxylated CNTs, in which many more new states are formed above the top of the valence band. Thus, functionalization with -OH and COOH groups transforms carbon nanotubes into electrodes that have a high capacity to oxidize electroactive species.

### 2.3. ORR, HER, and OER

**Figure 6:**
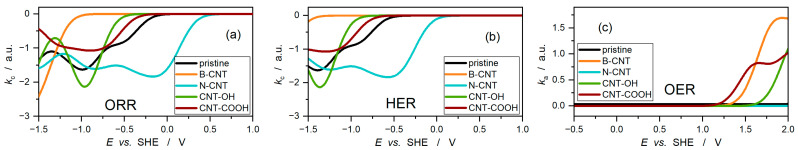
(**a**,**b**) are cathodic reaction rate curves, $k_{c}$, of the ORR and HER calculated for pristine, heteroatom-doped, and functionalized CNTs. (**c**) Anodic reaction rate curves, $k_{a}$, for OER for pristine and modified CNTs.

The curves of cathodic ORR and HER on pristine, heteroatom-doped, and functionalized CNTs are compared in Figure 6a,b. The curves reflect the role of the conductivity quasi-band in the performance of the cathodic reaction. Acceptor doping induced through the substitution of carbon atoms with boron atoms shifts the conductivity quasi-band of the CNTs toward higher energies (Figure 4a), resulting in the absence of electron states that could participate in electron transfer from the electrode to O_2_ (ORR) and H^+^ (HER). Therefore, the onset of the cathodic reaction on the B-CNT electrode is shifted toward lower potentials by as much as 0.9 V compared to the pristine CNT electrode. The reverse is true for acceptor-doped nanotubes. By shifting the conduction quasi-band downward in N-CNTs (Figure 4b), more electron states from the conduction band can participate in electron transfer. Therefore, the onset of the cathodic reaction is shifted toward higher potentials of the N-CNT electrode by 0.5 V compared to pristine CNT.

Functionalization with -OH and -COOH groups leads to only a slight shift of the conduction quasi-band edge toward higher energies (Figure 5a,b). The onset of the ORR and HER shifts toward lower potentials by only 0.2 V in CNT-COOH and by 0.4 V in CNT-OH relative to pristine CNT.

On the other hand, states from the quasi-valence band contribute to the performance of the anodic OER, which is shown in Figure 6c. Due to the high standard potential of OER (1.23 V vs.  SHE), pristine CNTs show negligible OER catalytic activity. Even more overstimulating is the introduction of donor dopant, which has the effect of shifting the top of the valence band to lower energies (Figure 4b). For this reason, in the $k_{a}$ curves of pristine CNTs and N-CNTs, the onset of the anodic reaction is not observed in the range of the calculated electrode potentials. In contrast, any modification of the electron structure leading to the formation of additional electron states in the valence quasi-band contributes to lowering the onset of the OER reaction. As we show in Figure 6c, substitution of carbon atoms by boron is very effective. Also, functionalization with -OH and -COOH groups contributes to lowering the OER onset potential, with the carboxylation of nanotubes yielding better results.

## 3. Discussion

There are a number of tools to track doping- and functionalization-induced changes in the structure of sp^2^ carbons, such as X-ray fluorescence spectroscopy (XPS), Fourier transform infrared (FTIR) spectroscopy, or Raman spectroscopy. Based on the XPS results, both the presence of the binding states, which correspond to the specific functional groups, can be identified [35], and the heteroatom content can be assessed [20]. XPS is used for surface chemical characterization of materials, so analytical results are often affected by impurities in the form of hydrocarbons and oxides permanently adsorbed from the air. While FTIR is an invaluable technique in identifying chemical functional groups in bulk carbon nanoforms [39], it does not provide much information about the properties of sp^2^-bonded aromatic carbon backbone. Raman spectroscopy is the most versatile spectroscopic technique when it comes to assessing the effects of heteroatom doping and functionalization on the electronic structure and, consequently, on the electrocatalytic properties of sp^2^-bonded carbon nanoforms. From Raman spectra, information on the dimensionality of carbon nanoforms, the concentration of defects, and the concentration of holes or electrons can be extracted simultaneously. This is an essential advantage over XPS and FTIR, which, in practice, should be used as a complement to Raman spectroscopy.

**Figure 7:**
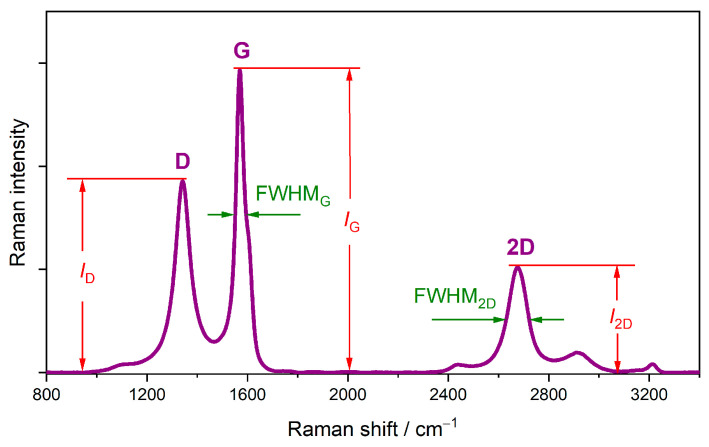
Raman spectrum of partially reduced graphene oxide, showing how to determine such parameters as $I_{D}$, $I_{G}$, $I_{2D}$, and FWHM.

In Figure 7, we show an example spectrum of partially reduced graphene oxide, where the main Raman features of the sp^2^-bonded carbons are highlighted. The D band at ~1340 cm^−1^ is the first-order hexagon breathing mode, and it is inactive in a perfect lattice. The G band at ~1580 cm^−1^ comes from the in-plane C-C bond stretching vibrations of a hexagonal carbon lattice. The 2D band at ~2680 cm^−1^ is the second-order hexagon breathing mode [61,62].

The $I_{D}/I_{G}$ ratio delivers information about the disorder. In the case of point defects, it is possible to estimate the defect density from $I_{D}/I_{G}$ [63,64]. The presence of intrinsic defects also causes a decrease in 2D band intensity. This allows the crystalline quality to be assessed on the basis of the $I_{2D}/I_{G}$ ratio. Additionally, doping results in increasing the full width at half maximum (FWHM) of all Raman features. Electron and hole doping shifts the position of the G band to higher frequencies [65]. The position of the 2D band allows us to distinguish between electron and hole doping. Upon increasing the charge transfer into the π* band (donor doping), the G mode softens, and the position of the 2D band shifts two frequencies lower. An opposite effect produces the introduction of holes into the π band; the 2D mode stiffens, and the 2D band shifts to higher frequences [65,66,67].

The correlation between intrinsic defects and HET has been studied in graphene produced through laser beam irradiation of carbon precursors [68]. Graphene obtained through irradiation with a UV laser beam showed a lower $I_{2D}/I_{G}$ ratio than graphene produced using an IR laser beam. The lower crystalline quality of the graphene translated into enhanced HET kinetics. For both Fe(CN)_6_^3−/4−^ and Ru(NH_3_)_6_^3+/2+^ redox couples, the HET standard rate constant was about twice as high on the UV-produced graphene with more intrinsic defects. In turn, one paper [69] reported a strong correlation between the $I_{D}/I_{G}$ ratio and the improved HET kinetics manifested by reduced redox peak separation of cyclic voltammograms of the Fe(CN)_6_^3−/4−^ redox couple, which was observed in reduced graphene oxides. A comprehensive study on the effect of point defects in graphene generated by Ar^+^ irradiation on electrocatalytic activity is provided by Ref. [70]. In that study, FcMeOH was used as an electrochemical probe. The standard rate constant increased by more than one order of magnitude when the point defect density estimated from $I_{D}/I_{G}$ increased from $4.2\times 10^{10}$ to $5.7\times 10^{12}\;{cm}^{-1}$.

The influence of heteroatom doping on electrocatalytic activity was investigated in boron- and nitrogen-reduced graphene oxides through electrochemical impedance spectroscopy [71]. Impedance spectra of the Fe(CN)_6_^3−/4−^ redox couple show a significant decrease in electron transfer resistance for both N-doped and B-doped graphene, with a greater decrease occurring for acceptor doping. This behavior is explained in Figure 3. An upward shift of the electron bands (boron doping) is more conducive to electron transfer than a downward shift of the bands (nitrogen doping) because the Fermi level of the redox couple, $\epsilon_{F,redox}$, is below the Dirac point (the Fermi level of pure graphene, Figure 2). We might expect that for a redox couple with a higher $\epsilon_{F,redox}$, (lower standard potential), the donor dopant would be more effective.

A similar effect is produced by heteroatom doping of carbon nanotubes. On boron-doped CNTs, the HET kinetics of Fe(CN)_6_^3−/4−^ is slightly better than on nitrogen-doped CNTs and much better than on pristine CNTs [72]. In both of the case studies referred to, the carbon sp^2^ network was heavily defected, so the subtle G-band shift phenomenon in the Raman spectra could not be observed.

A strong influence of covalent functionalization with -OH group on the HET kinetics has been reported in Ref. [35]. Hydroxylation of carbon nanotubes results in enhanced HET kinetics between the Fe(CN)_6_^3−/4−^ redox couple and the electrode, whereas for the Ru(NH_3_)_6_^3+/2+^ redox couple the HET standard rate constant decreased radically. Raman spectra of pristine CNTs and OH-CNTs have confirmed the effect of donor doping, and a blue-shift of the G- and 2D-band has been observed.

In Ref. [73], it has been shown that strongly N-doped CNTs synthesized from ethylenediamine are an excellent catalyst of ORR, which manifests itself in a shift of the ORR onset potential toward higher potential and a significant reduction of the current density compared to pristine CNTs. Strong donor doping is confirmed in that work by the shift of the G-mode to lower frequencies. The enhanced HER performance of N-CNTs, relative to pristine CNTs, was in turn shown in Ref. [74], where donor doping is also confirmed by Raman data. On the other hand, enhanced catalytic activity of B-CNTs toward OER has been reported in [75], where a reduction in the OER onset potential and a marked increase in the anodic current density on the B-CNT electrode relative to pristine CNTs were observed. The positive effect of carboxylation of nanotubes on OER performance is reported in Ref. [76]. In that work, it was shown that carboxylation of the nanotubes caused a downward shift in the onset potential of the OER by 0.4 V.

## 4. Materials and Methods

The π-electron DOS of graphene was calculated using the nearest neighbor TB model [77,78], where zero energy is referenced as the Fermi level, $\epsilon_{F}$. Calculation of graphene’s DOS with structural defects was based on the assumption that disorder induces a resonant impurity band near the Fermi level and that it is characterized by the Thomas–Porter distribution [79]. In the calculation of graphene’s DOS subjected to substitutional doping with acceptor or donor atoms on the DOS, we relied on the assumption that holes or electrons injected into the graphene lattice by acceptor or donor dopants cause a shift of the Dirac point by $\delta \epsilon_{DP}=\pm \sqrt{\pi }\hbar v_{F}\sqrt{n}$. Here, the plus sign refers to acceptor (hole) doping, while the minus sign refers to donor (electron) doping. The Fermi velocity $\hbar v_{F}=6.73\;eV\;Å$ is an experimental parameter taken from the literature, and n is the carrier concentration (holes or electrons) [80].

The electronic structure of carbon nanotubes was studied through density functional theory (DFT). A pristine nanotube CNT(5) made of 420 carbon atoms was considered as an initial model. We used the ωB97XD [81] exchange-correlation functional and 6-31G(d) basis set, which has previously proven to be a reasonable combination for studying the electron structure of large organic systems [82,83,84]. Numerical simulations were carried out with the Gaussian16 computational package [85]. The nanotube’s DOS was extracted from Gaussian output data by using GaussSum software (Version 3.0, https://gausssum.sourceforge.net/, accessed on 7 March 2025) [86].

The cathodic reaction rate, $k_{c}$, and the anodic reaction rate, $k_{a}$, on an electrode with a specific DOS were calculated as a function of electrode potential, E, using the Gerischer–Marcus integrals [87,88,89]:

(1a) $$k_{c}\left(E\right)\propto \int_{-\infty }^{\infty }f(\epsilon -eE)\;DOS(\epsilon -eE){\;W}_{ox}(\epsilon )\;d\epsilon ,$$

(1b) $$k_{a}\left(E\right)\propto \int_{-\infty }^{\infty }[1-f(\epsilon -eE)]\;DOS(\epsilon -eE){\;W}_{red}(\epsilon )\;d\epsilon .$$

Equation (1a) describes the HET of electrons from the occupied states expressed as the product of the Fermi–Dirac distribution, f(ϵ−eE), and the density of electronic states, DOS(ϵ−eE), to the unoccupied states of the oxidized form of the redox couple, ${\;W}_{ox}(\epsilon )$. In the anodic reaction, electrons are transferred from filled states of the reduced form of the redox couple represented by ${\;W}_{red}(\epsilon )$ to empty states of the electrode given by the product of [1−f(ϵ−eE)] and DOS(ϵ−eE).

Distribution of the unoccupied redox states of the electron acceptor (the oxidized form of the redox couple) and the occupied states of the electron donor (the reduced form of the redox couple) in the electrolyte is given by Gaussian distributions:

(2a) $$W_{ox}\left(\epsilon \right)=\frac{1}{4\pi \lambda kT}\exp\left(-\frac{{\left(\epsilon -\epsilon_{F,redox}-\lambda \right)}^{2}}{4kT\lambda }\right),$$

(2b) $$W_{red}\left(\epsilon \right)=\frac{1}{4\pi \lambda kT}\exp\left(-\frac{{\left(\epsilon -\epsilon_{F,redox}+\lambda \right)}^{2}}{4kT\lambda }\right),$$

where $\epsilon_{F,redox}$ is the Fermi energy of the redox species obtained through the conversion of its standard potential to an absolute energy scale and λ is the reorganization energy of the redox species. Standard potentials of Fe(CN)_6_^3−/4−^ and Ru(NH_3_)_6_^3+/2+^ are +0.37 V and +0.1 V vs. SHE, respectively [90]. Their equivalent values on the energy scale are −4.87 eV and −4.6 eV, respectively. Reorganization energies, λ, of the Fe(CN)_6_^3−/4−^ and Ru(NH_3_)_6_^3+/2+^ redox probes are 0.35 eV and 0.8 eV, respectively [91]. In calculating the $k_{c}\left(E\right)$ of ORR and HER, we assumed the values of standard reduction potentials of 0.402 V vs. SHE and 0.0 V vs. SHE [58], which on the energy scale correspond to values of −4.902 eV and −4.5 eV vs. vacuum, respectively. The $k_{a}(E)$ of OER was calculated assuming a standard reaction potential of 1.23 V ($\epsilon_{F,redox}=-5.73\;eV$). In the calculation of ORR, HER, and OER rates, a value of λ=0.26 eV was assumed [92]. HET through the nanocarbon electrode–electrolyte interface depends on alignment between the Fermi level of the nanocarbon electrode, $\epsilon_{F}$, and the distribution of the electron states of redox species in the electrolyte [11,12,93,94]. In calculations, the $\epsilon_{F}$ in graphene and carbon nanotubes was set to −4.5 eV. It is an averaged value derived from the work function of graphene and various nanotubes [95].

## 5. Conclusions

Upon analyzing the cathodic and anodic reaction curves, we draw the following conclusions, which also provide guidance on how to match nanocarbon electrodes to specific electrochemical reactions:

1. If we want both anodic and cathodic reactions to occur at lower electrode potentials, we use acceptor-doped nanocarbons as the electrode material. This is particularly true for OER, which should start at the lowest possible anode potential;
2. If we want both anodic and cathodic reactions to occur at higher electrode potentials, we use donor doping. This guideline is particularly applicable to ORR and HER catalysis, where we are concerned that the onset of the ORR and HER reaction should occur at the highest possible cathode potential;
3. If we want the anodic reaction to primarily occur at lower electrode potentials, we use functionalized nanocarbons (functionalization with a -COOH group is more effective than -OH). This tip, in particular, is applicable to OER catalysis;
4. If we do not care about the selectivity of the reaction and focus only on the efficiency, we use electrodes with intrinsic defects (point defects, edges, etc.).

Indeed, the above rules apply to Fermi level engineering of sp^2^-bonded nanocarbons, which is an effective tool for matching the electrode material to the redox potential of a specific electroactive medium. This translates into all possible electrochemical applications, including electrochemical sensors, fuel cells, metal–air batteries (ORR), and electrochemical water splitting (HER and OER). Referring to the selected case studies, we show that the doping effect can be easily monitored in nanocarbons using Raman spectroscopy.

## Data Availability

The data presented in this study are available from the corresponding author upon reasonable request.

## Abbreviations

The following abbreviations are used in this manuscript:

HET	Heterogeneous electron transfer
DOS	Density of states
CNT	Carbon nanotube
SHE	Standard hydrogen electrode
ORR	Oxygen reduction reaction
HER	Hydrogen evolution reaction
OER	Oxygen evolution reaction
